# Applying the Toyota production system to decrease the time required to transport patients undergoing surgery from the general ward to the operating room and reviewing the essence of lean thinking

**DOI:** 10.3389/fmed.2022.1054583

**Published:** 2022-12-07

**Authors:** Chien-Chung Lin, Jui-Wen Chueh, Hung-Ming Chen, Yao-Hui Chiu, Dachen Chu

**Affiliations:** ^1^Department of Orthopedic Surgery, General Education Center, Taipei City Hospital, University of Taipei, Taipei City, Taiwan; ^2^Department of Quality Management, Taipei City Hospital, National Taipei University of Nursing and Health Sciences, Taipei City, Taiwan; ^3^Department of Orthopedic Surgery, Taipei City Hospital, Taipei City, Taiwan; ^4^Department of Anesthesia, Taipei City Hospital, Taipei City, Taiwan; ^5^Department of Superintendent, Taipei City Hospital, School of Medicine, College of Medicine, Institute of Public Health, National Yang Ming Chiao Tung University, Taipei City, Taiwan

**Keywords:** lean thinking, operating room, Toyota production system, value stream map, transportation, continuous improvement, quality, patient safety

## Abstract

**Background:**

Sending a patient to the operating room is the first step in surgery. Delayed patient transport causes the patient to go hungry for a longer time, aggravating the patient’s physical discomfort and psychological stress. The issue of delays in transporting inpatients to the operating room has rarely been discussed in the literature. The Toyota production system is a famous and excellent scientific method of reducing waste and increasing efficiency. Our goal is to use the Toyota method to decrease the time required to transport the inpatient to the operating room and to review the concepts underlying lean thinking.

**Methods:**

We employed an A3 8-step problem-solving process. A current value stream map featuring numerical data (concerning 46 patients) measured in the workplace was developed. The total time spent on transport was 53 min, but we expected patients to be transported within 30 min. We hoped to reduce the time wasted by half, i.e., by 23*50% = 12 min. These 12 min were saved by reducing the time spent on “waiting for an attendant at the ward” by 9 min and the time spent on “elevator transport” by 3 min. According to the value stream map featuring the time measurements, the root causes of delayed transportation can be divided into process-related, attendant-related, and elevator-related factors. We formulated 5 countermeasures. The ECRS (Eliminate, Combine, Rearrange, Simplify) technique was used to rearrange, combine, and simplify the existing process. Hospital executives established norms for attendant prioritization of work and rules for elevator use.

**Results:**

According to the original indicators, all goals were attained. “Total time spent” decreased by 62.3%. The time required for attendants to report to the nursing station decreased by 56.5%. The time spent on elevator transport decreased by 44.4%. We developed a process for future use based on information-assisted patient and staff identification. Finally, we standardized successful processes.

**Conclusion:**

The seemingly trivial factors that delay patient transport are associated with seven types of waste. The A3 8-step problem-solving process is useful in this context. In proposing this improvement process, we believe that we are following the spirit of the Toyota production system.

## Introduction

Few patients can be the first to undergo surgery in the operating room (OR) in a day. Most patients must wait for surgery, especially patients who are last in line to undergo surgery, who may have to wait for more than 6 h. The longer the patient’s wait is, the less safe the situation is for the patient and the higher the risk of surgery and anesthesia. Waiting for surgery in the general ward represents a kind of mental torment for patients and their families, and all patients hope to undergo surgery sooner. Sending a patient to the operating room for surgery is the first step in the process, and sending the patient to the operating room as soon as possible has high value for patients and their families.

In our hospital, because doctors and operating room staff often had to wait for the patient to travel from the ward to the operating room, most doctors and colleagues believed that “the ward is too slow to send patients to the operating room.” Although the operating room management committee of our hospital has established a rule mandating that “the patient with the next operation will be sent to the operating room 30 min before the currently undergoing surgery is completed,” the staff still believed that “the ward is too slow to send the patient.” One physician waited 50 min for his patient to be sent to the operating room and angrily reported an abnormal event via the hospital’s abnormal notification system in person. If a problem is defined as a gap between our expectations (the patient being sent to the OR more quickly) and the current state of affairs (the patient being sent to the OR too slowly), this situation represents a problem. In actual society, only problems that have bad effects must be solved. The highest priority that must be addressed in the healthcare system is related to patient safety and quality of medical care. Therefore, taking the patient to the OR too slowly is a real problem in the healthcare system that must be addressed.

We hoped to find a solution by conducting a literature review. After a thorough literature review, it was found that the topics with which all medical-related personnel were concerned pertain to OR productivity and efficiency and whether the first operation begins on time (1–5). The topic or issue of delays in transporting inpatients to the operating room has rarely been discussed in the literature. Because the Toyota production system (TPS), which frequently refers to the notions of lean management or lean thinking, is a good and scientific method for reducing waste and increasing efficiency (6, 7), we decided to apply a TPS approach to reduce the time required for inpatients to travel to the operating room. Our short-term goal was to eliminate 50% of the time wasted sending patients to the OR over a period of 6 months.

## Materials and methods

We organized a new team. The team members included the chief of surgery, the director of the OR, the head nurse of the OR, two OR nurses, the head nurse of the ward, two ward nurses, an anesthesiologist, a project manager, and a surgical specialist nurse. The lean project was supported and approved by the OR management committee and the hospital director. We bought books on the topic of lean management for study. Our team attended an 8-h lean management workshop to study the concept of lean thinking and acquire some lean tools. One month later, we spent 2 h asking a lean expert to guide us to determine whether the tools e used were correct and whether the value stream map we developed was realistic and executable. We followed the advice of this teacher by employing an 8-step problem-solving process (A3 management process, A3 problem-solving, A3 document) to address the problem we sought to resolve (8–10). The 8 steps of this process are as follows: the first step is to clarify the problem, the second step is to break the problem down, the third step is to establish a target, the fourth step is to analyze the root cause of the problem, the fifth step is to develop countermeasures, the sixth step is to implement those countermeasures, the seventh step is to evaluate both the results and the process, and the eighth step is to standardize and share successful processes (8–10). To ensure continuous improvement, these 8 steps must consistently be flowing and recursive.

### The first step is to clarify the problem

**FIGURE 1:**
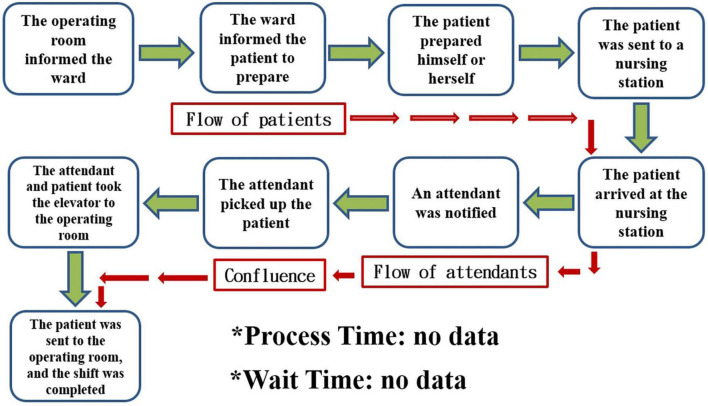
The entire flow of inpatients who are sent to the operating room in the existing process.

Our question was “Is the process of transporting patients from the general ward to the OR truly too slow?” Although most physicians and OR staff agreed that the ward was too slow to send patients to the OR, we required numerical data to support this claim. The entire flow of inpatients being sent to the operating room according to the existing process is shown in Figure 1.

We defined “the time required for the patient to be sent to the operating room” in terms of the “the time the patient arrived at the OR” minus “the time the OR informed the ward to send the patient.” The time is measured in minutes. Because our hospital’s information system does not record the time required to notify the patient that he or she is being sent to the OR, we did not have accurate data regarding the amount of time it takes for the patient to arrive at the OR. To obtain such data, we had to visit the scene (i.e., the workplace) of the procedures by which the patient is sent to the OR to take observations and measurements (6, 11–13). We employed a patient-centered approach to observe patient movement and record the time points of patient movement, so the factor we measured was mainly “the flow of patients.” The recorded time points associated with the patient’s movement included the following: the time the patient received the notification, the time the patient prepared himself or herself, the time the patient arrived at the nursing station, the time the attendant received the patient, and the time the patient arrived at the OR. A total of 20 patients were measured (Table 1). According to the time measured in Table 1, we could calculate the time spent on each action (Table 2).

**TABLE 1:** The actual time spent in the workplace measured in hours and minutes.

	Measured time				
Patient number	What time was the patient notified?	What time was the patient ready?	What time did the patient arrive at the nursing station?	What time did the attendant receive the patient?	What time did the patient arrive at the operating room?
1	07:05	07:15	07:17	07:40	07:52
2	07:10	07:16	07:20	07:50	07:59
3	07:10	07:20	07:25	07:50	07:56
4	07:30	07:38	07:45	08:20	08:26
5	09:15	09:22	09:25	09:53	10:00
6	09:38	09:44	09:48	10:21	10:30
7	10:25	10:30	10:33	10:48	11:16
8	11:15	11:25	11:30	11:54	12:14
9	11:30	11:41	11:50	12:23	12:29
10	12:25	12:34	12:42	12:56	13:06
11	12:40	12:55	13:05	13:21	13:31
12	13:20	13:35	13:37	14:08	14:17
13	13:40	13:46	13:50	14:19	14:28
14	07:15	07:30	07:35	07:55	08:08
15	08:20	08:35	08:38	08:52	09:00
16	09:50	09:58	10:05	10:17	10:29
17	11:30	11:37	11:41	11:46	11:57
18	08:25	08:40	08:48	09:05	09:09
19	08:45	08:57	09:05	09:27	09:33
20	09:45	09:57	10:05	10:31	10:34

**TABLE 2:** The amount of time spent is calculated based on Table 1.

	Time spent (in minutes)				
Patient number	Time spent allowing the patient to become ready	Time spent traveling to the nursing station	Time spent waiting for an attendant to report to the ward	Time spent traveling from the ward to the operating room	Total amount of time spent
1	10	2	23	12	47
2	6	4	30	9	49
3	10	5	25	6	46
4	8	7	35	6	56
5	7	3	28	7	45
6	6	4	33	9	52
7	5	3	15	28	51
8	10	5	24	20	59
9	11	9	33	6	59
10	9	8	14	10	41
11	15	10	16	10	51
12	15	2	31	9	57
13	6	4	29	9	48
14	15	5	20	13	53
15	15	3	14	8	40
16	8	7	12	12	39
17	7	4	5	11	27
18	15	8	17	4	44
19	12	8	22	6	48
20	12	8	26	3	49
Median	10	5	23	9	48

The longest case measured required 59 min to transport the patient to the OR, the shortest required 27 min, and the median was approximately 48 min. We expect that when the OR informs the ward that the patient should be sent to the OR, the patient should be sent to the OR within 30 min. However, an average of 48 min was required for patients to be sent to the OR. Because there was a gap between our expectation and the current state of affairs and due to the fact that sending patients to the OR too slowly can affect patient safety and the quality of medical care, the problem of excessively slow transfer must indeed be solved.

### The second step is to break the problem down

**FIGURE 2:**
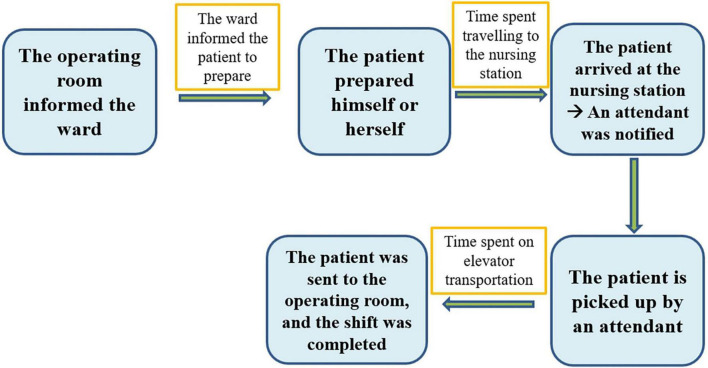
Value stream map of the existing process.

**FIGURE 3:**
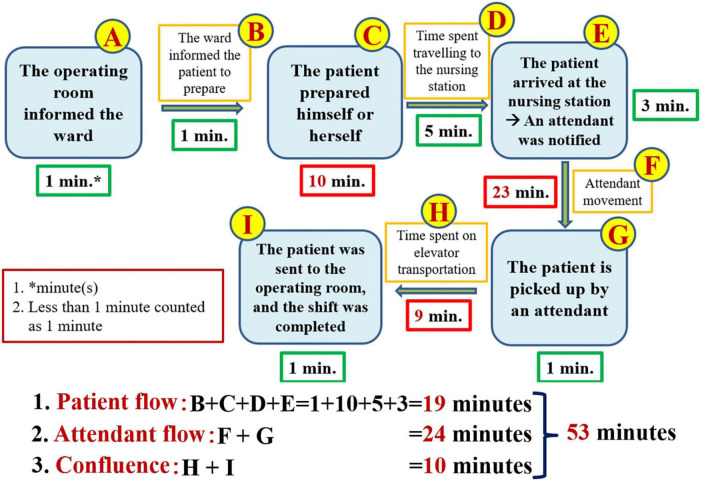
Value stream map including the amount of time measured before improvement.

To break the problem down, constructing a value stream map is an indispensable step. The proper way to construct a value chain diagram is to depict the flow of the entire transportation process clearly (Figure 1) and then to focus on the part of this process that is valuable (value-added) to the patient (7, 8, 11). The following figure displays the value stream map of the existing process (Figure 2). Recording the time spent on the value stream map for the existing process, we can see that it took 53 min for the patient to arrive at the operating room (Figure 3).

As shown in the value stream map, 53 min (median) was the total amount of time we measured, but we expected patients to be delivered within 30 min. The 23-min difference in the median indicates the problem, i.e., waste. The most time-consuming steps in this process are, in order, the time spent on attendant travel (23 min), the time spent on patient preparation (10 min), and the time spent in elevator transportation (9 min). When we discovered that notifying attendants to bring patients to the OR was the most time-consuming bottleneck, we began to reorganize our team and added the attendant head nurse and 3 attendants to our team. For the purposes of this article, hospital attendants must complete patient-related work, general ward-related work and temporary assignments. The patient-related content of the job of an attendant involves helping patients or transporting them to places such as the OR, the radiological department, the ultrasound room, the rehabilitation room, and the dialysis room, where patients can receive treatment or undergo examinations. The general ward-related content of the job of an attendant entails borrowing records from the ward and returning medical records, helping collect medicines, medical-related items, blood products, and patient-related samples, and returning unused items. The job contents of temporary assignments are temporarily decided by the head nurse of the ward and a nurse practitioner. We communicated with attendants regarding the concepts of patient-centered care and lean management. To ensure respect for the feelings of the attendants, we told them that we cared about the process, the content and quantity of their work, and the safety and care quality of patients. We did not discuss the issue of their own abilities, qualifications, and diligence. We did not condemn any colleagues who did not meet the appropriate standard.

### The third step is to establish a target

We hoped to reduce the amount of wasted time by half (50%), 23*50% = 12 min, so our short-term goal was to reduce the total time spent on this process by 12 min and to ensure that the patient would be sent to the operating room within 41 (= 53–12) min. How can the 12 min to be eliminated be allocated? We hoped to reduce the time spent “waiting for an attendant to arrive at the ward (attendant movement)” by 9 min and the time required for “elevator transport” by 3 min. We defined achievement rate in terms of the number of patients arrived at the OR in less than 41 min divided by the number of all patients. We hoped that the achievement rate would be greater than 80%. The time (10 min) that the patient spends preparing himself or herself for surgery is value-added to the patient, so no decrease in this time is intended for the time being.

### The fourth step is to analyze the root cause

**FIGURE 4:**
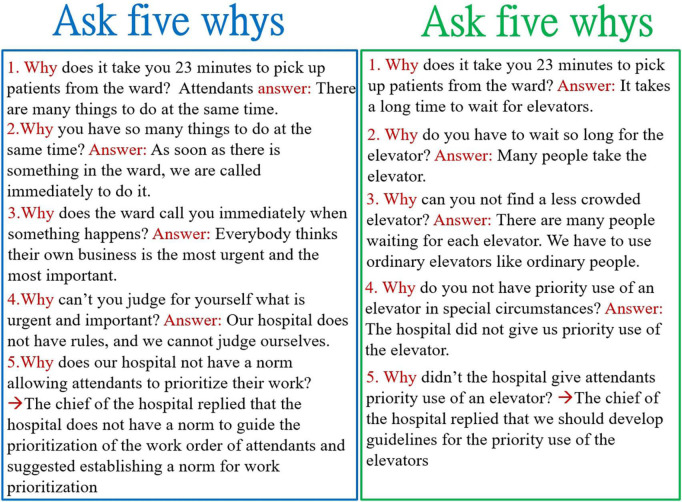
Five why questions were used to identify the root cause of attendant-related problems.

According to the value stream map featuring the measured time, which is presented in Figure 3, the possible causes of delayed transportation that should be solved can be divided into process-related (see the patient flow and attendant flow), attendant-related, and elevator-related causes. We created a fishbone diagram to uncover the main causes of our problem (not shown). We asked five why questions to discover the root causes of the problem (6). The following is a demonstration of the power of asking the question of why 5 times to discover the root cause of attendant-related problems (Figure 4).

### The fifth step is to develop countermeasures, and the sixth step is to implement those countermeasures

**FIGURE 5:**
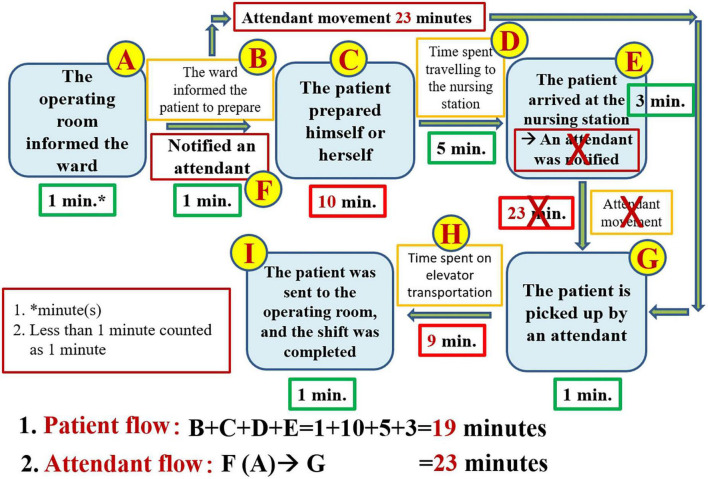
Rearrange the step of notifying attendants.

**FIGURE 6:**
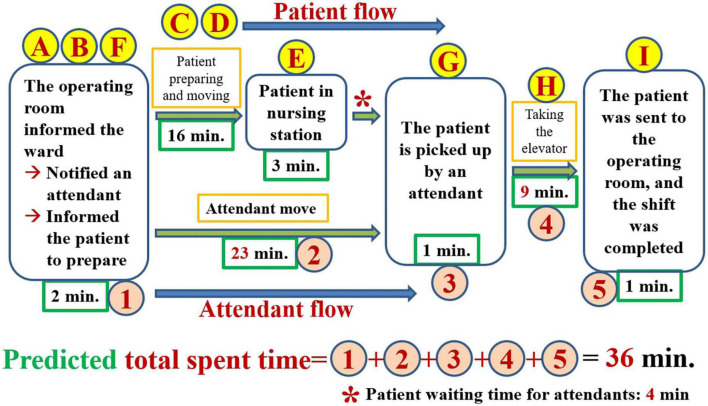
The improved value stream map after combining and simplifying the rearranged process shown in Figure 5.

Based on the results of the root cause analysis, we formulated 5 countermeasures, which are illustrated in Table 3. The ECRS (Eliminate, Combine, Rearrange, Simplify) technique (14) was used to analyze the existing process. The highest priority in developing countermeasures is to change the process for notifying attendants. In Figure 3, we can see that the “patient flow” took a total of 19 min for the path BCDE, while the attendant flow took 23 min. As long as the attendant is “notified” as soon as the operating room informs the ward, the 23 min of the attendant flow can overlap with the 19 min of the patient flow. Therefore, simply by rearranging the process to change when the attendants are notified, i.e., notifying attendants in advance, can be predicted to decrease the 23 min that patients currently spend waiting for the movement of the attendant to 4 min (= 23–19) in the future (Figure 5). When we further combine and simplify the rearranged process in Figure 5, the value stream map after improvement becomes more concise and easier to understand (Figure 6).

**TABLE 3:** Formulate and implement countermeasures.

Problem (Priority)	Root cause	Countermeasures	Implementation	Expected outcome
Process-related (1)	Notify attendants too late.	Change the process for notifying attendants.	Notify attendants when the ward nurse informs the patient to prepare himself or herself for transportation to the operating room.	The time spent waiting for attendants to move patients can be reduced by 19 min (from the time when the ward notifies the patient to the time the patient arrives at the nursing station)
Attendant-related (1)	1. Call the attendant as soon as there is anything in the ward. 2. A great deal of work must be done simultaneously without classification. 3. Too busy to support each other.	1. Organize and categorize the work content of the attendants (5S). 2. Establish the order in which attendants should prioritize their work.	1. Use the attendant daily task time login form. 2. Organize and categorize the attendants’ work content 3. Establish the order in which attendants should prioritize their work.	1. The time spent waiting for attendants to arrive at the ward can be reduced by at least 9 min. 2. The total amount of time spent can be reduced by at least 9 min.
Elevator-related (2)	No guidelines for the priority use of the elevator.	Formulate guidelines for the priority use of the elevator.	1. The whole hospital announces the guidelines for the priority use of the elevator 2. Use color and text to indicate events of the 1^st^ and 2^nd^ levels of urgency.	1. the amount of time spent waiting for the elevator to arrive at the ward can be reduced by at least 3 min. 2. The total amount of time spent can be reduced by at least 3 min.
Elevator-related (2)	No staff-only elevator available.	Designate an elevator a staff-only elevator.	Coordinate the use of the special elevator across disciplines.	Same as above.

Attendant-related issues are the second highest priority in developing countermeasures. To increase the speed with which the attendant transports patients to the OR, we must focus on the content and quantity of the attendant’s work. We visited the general ward once again to convey that the ward must do what the hospital originally stipulated for 5S to reduce waste in staff movement and time spent waiting. The hospital stipulates that attendants should collect certain non-emergency objects (such as specimens, discharge medicines, inspection sheets, etc.) only at a specific time (every hour) to reduce the number of times that attendants must be called to the ward to deal with such issues. Hospital executives also establish norms for the prioritization of work by attendants. The top priority for attendants’ work is providing all necessary intensive care unit-related and emergency-related medical care. Non-emergency events related to the OR are the second priority. Hospital executives develop guidelines for elevator use according to which one of the hospital elevators is chosen for use as a dedicated elevator for top priority and second-level priority events. Moving a hospital bed into or out of the operating room is classified as a second-level priority.

## Results

### The seventh step is to monitor both the results and the process

According to the value stream map after improvement, it was necessary to return to the workplace to take more observations and measurements to investigate whether our countermeasures were effective. The time points for measuring patient movement, including the time at which the patients and attendants were notified, the time the patient arrived at the nursing station, the time the attendant arrived at the nursing station, the time the patient was given into the custody of the attendant, and the time the patient arrived at the operating room, are shown in Supplementary Figure 1. The calculation of the time required for each action is also shown on the right side of Supplementary Figure 1.

**FIGURE 7:**
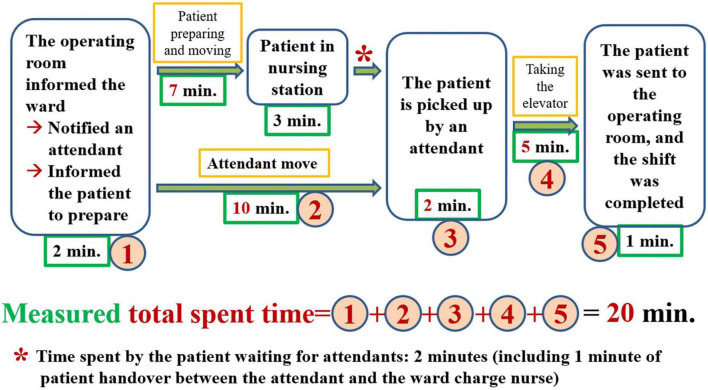
The value stream map after improvement including time measurements.

By incorporating the calculated amount of time spent into the value stream map after improvement, we can see that it took 20 min (median) for the patient to arrive at the operating room (Figure 7). The time spent by the patient waiting for an attendant to bring her or him to the OR was 2 min, including 1 min of patient handover between the attendant and the charge nurse of the ward.

**FIGURE 8:**
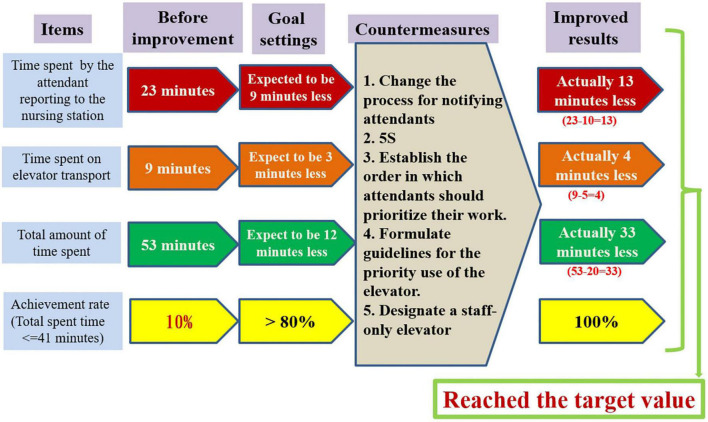
Comparison of the results before and after the improvement.

We originally developed 4 indicators (Figure 8). After implementing the 5 countermeasures, all indicators reached the goals. “Total time spent” decreased by 62.3%. If achievement rate is defined in terms of the number of patients who took patients who arrived at the OR in less than 41 min divided by the number of all patients, then the achievement rate after improvement was 100% (see Supplementary Figure 1). The time spent by attendants reporting to the nursing station decreased by 56.5%. The time spent on elevator transport decreased by 44.4%.

### Continuous improvement is an important way of thinking that must employed and constantly remembered

**FIGURE 9:**
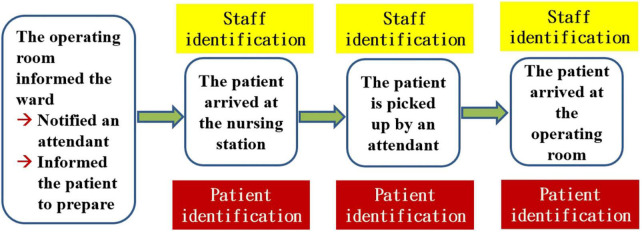
The future process based on computer information-assisted patient and staff identification.

In the context of patient transport to the OR, “patient identification” and “staff identification” are very important steps for every hospital. Our hospital features barcode machines that are used in the ward and the OR to identify medical-related materials. If we can use the barcode machine to assist with patient and staff identification or if these identifications can rely on computer information technology in the future, the input of the time reaching a check point can be recorded electronically and thus made more accurate. Therefore, we believe that we can use “the points that patients and staff need to be identified” to simplify this process further. For example, “The patient arrived at the nursing station,” “The patient was picked up by an attendant” and “The patient arrived at the operating room” all require patient and staff identification. Figure 9 shows our future process based on computer information-assisted patient and staff identification.

Because the process was revised once again, we returned to the workplace to confirm the process. Data regarding the time points for measuring patient movement, including the time at which the patients and attendants were notified, the time the patient arrived at the nursing station, the time the patient was picked up by an attendant, and the time the patient arrived at the OR, are shown in Supplementary Figure 2. The calculation of the time spent on each action is also shown in the right side of Supplementary Figure 2.

**FIGURE 10:**
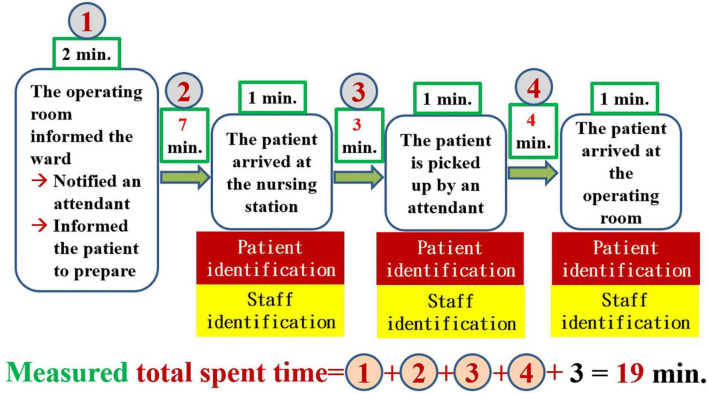
The future process based on computer information-assisted patient/staff identification with time measurements.

Including the information regarding the time spent (Supplementary Figure 2) in the future process based on patient/staff identification, we can see that 19 min (median) were required for the patient to arrive at the operating room (Figure 10). The time spent by the patient waiting for an attendant to transport her or him to the OR was 3 min, including 1 min of patient handover between the attendant and the charge nurse of the ward. The achievement rate (= 41 min) was 85.0%. The test results all meet our previously specified target value. Accordingly, we can say that the future process presented in Figure 9 is appropriate and well-suited to meet additional needs.

### The eighth step is to standardize successful processes

**FIGURE 11:**
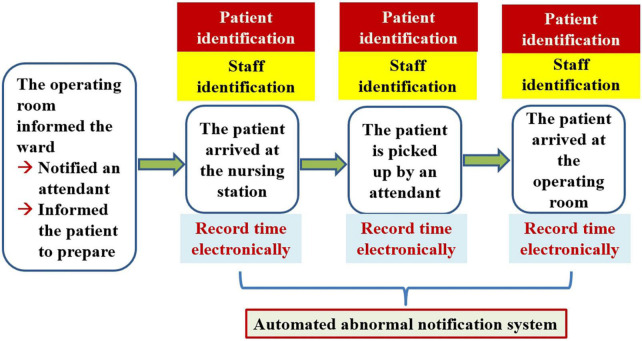
Final future process.

If the future process can accommodate the electronic identification of staff and patients, it will be easy in the future to use computer programs at any time to automatically send any abnormal signals to managers. Therefore, the combination of the future process with the automated abnormal notification system will constitute our final future process (Figure 11).

The task of standardizing successful processes includes process-related actions, attendant-related actions, and elevator-related actions. Process-related actions include the need to train every new general ward recruit in the processes associated with transporting patients to the OR and the recruitment of a computer language programmer to incorporate the time point of patient identification and staff identification and the time of notification to send the patient to OR into the electronic field of our hospital information system as a tracking item to measure the time spent transporting the patient to the OR. The final future process and the newly developed standard instructions are printed and posted in the most visible place in general wards (e.g., on the visual board). Attendant-related actions include the 5S and work priorities of the attendant, the education and training items required for new attendants in the general ward, and the task of ensuring that the attendants hand over their work items every month. Elevator-related actions include the creation of guidelines for the priority use of elevators and the communication of the guidelines regarding the use of special elevators throughout the whole hospital, including by posting these guidelines on the elevator bulletin board and their use in the context of a project related to the education and training of new recruits.

## Discussion

The problem with our OR lies in the fact that most physicians and nurses are unhappy with delays in the time spent transporting the patient from the ward to the OR. This fact causes patients to wait longer for surgery and to go hungry (consuming nothing by mouth) for a longer time, and it may cause instability in the patient’s physical and mental condition or require staff to work longer, resulting in overtime and staff fatigue. Choosing the appropriate approach to solve a problem is the first step in successful problem solving. The Toyota production system (TPS), which relies on lean management or lean thinking, considers waste (muda) to be the main cause of all problems or troubles. The basic purpose of TPS is to eliminate waste completely, and this process is intended to be implemented with the help of both the just-in-time principle and autonomation (jidoka, autonomous defect control) (6–8, 12). Just-in-time entails producing products when necessary and producing exactly the necessary quantity of products; that is, there is no waste. Pull production methods and Kanban systems have been developed to help achieve the goals of the just-in-time principle (6). Because the operating mode of the hospital’s OR requires that after one patient is finished, another patient is called into the OR for surgery, an approach which is suitable for a pull-type management system, and because the Kanban system used by hospitals on a daily basis governs the order in which patients undergo operations and establishes a reasonable number of surgeries per day, the management of our OR should be a suitable context for TPS (6–8, 13). As Taiichi Ohno (the developer of TPS) noted, teamwork is everything (6). Toyota’s culture is aimed at cultivating and respecting people (6, 8, 12, 13). To complete problem-solving tasks, we organize a cross-department group, and each team member has access to individual functions and technologies in his department. Our hospital allowed team members to attend a TPS course and search for a teacher. By learning, brainstorming, and problem-solving collaboratively, we can enhance the strength of our team.

According to the perspective of Toyota, problems are largely the result of a great deal of waste in the workplace (Gemba). Toyota’s point of view also claims that problems emerge from the workplace and that their solutions can thus be found in the workplace (6, 12, 13). A very important step is to go to the workplace with the aim of collecting all the relevant steps that we observe to create an entire process diagram (Figure 1). Because the patient is the most important person and the protagonist in the hospital setting, it is necessary to focus on the notion of added value for patients to create a value stream map that can be valuable to patients with respect to patient flow (Figure 2) (7, 8, 11). Creating the value stream map is a very important step in the task of decomposing the problem. Although thinking based on the Toyota perspective aimed at grasping the essence of problems, i.e., by focusing more on facts than numerical data, the use of data measured in the workplace to prove the facts is also an important scientific approach to this task (Table 1) (6–8, 12). If we use numerical data to highlight the problem, it becomes easier for everyone to agree on and be aware of the seriousness of the issue at hand. Therefore, it is more important to rely on numerical data to capture the problem than to rely on mere imagination.

Setting goals allows all members of the team to align themselves with the same targets and to focus on the most urgent and important tasks. Defining a target is similar to archery. Prior to shooting, you must find and aim at the target to hit the red point in the center. For beginners, the initial target setting value should neither be too high nor too low. The proper approach is to define a target that can be achieved with the application of some degree of effort and hard work. A deadline must also be established to ensure that the problem can be solved in a timely manner. The goals we set, i.e., the short-term goal of reducing the total time spent by 12 min over a period of 6 months, which included 2 specific targets (to reduce the time spent on attendant movement by 9 min and to reduce the time required for elevator transport by 3 min), should be appropriate, because the key point of this process is to ensure continuous improvement; as long as we, like a turtle, do not stop and continue to progress step by step, we can ultimately reach our destination (6). For skilled employees or organizations, goal setting should involve the establishment of goals that are difficult to achieve. As Womack and Jones noted in their book “Lean Thinking,” successful organizations set specific timetables for the achievement difficult goals and subsequently routinely meet or exceed those timetables, while low-achieving organizations merely set reasonable goals (7).

The root cause of a problem is similar to the roots of a weed. If the roots are not removed first in the process of eradication, the weeds continually grow back when the weather and environment improve. If the real cause of the problem is not solved, the same problem can recur. Only addressing the root cause of a problem can truly solve it. It can also be deduced that if a problem cannot be solved, this situation indicates that its root cause has not truly been found. In the culture of Toyota, after discovering the main causes of the problem (using the fishbone diagram), we can ask the question of why five times to uncover the root causes of the problem (6, 8). In some cases, it may be possible to find the root cause by asking the question of why only 2 or 3 times, but this limitation can easily result in the main cause rather than the root cause being found, and only continuing to ask the question of why 4 to 5 times allows the root cause of the problem to be found easily (6). How can we ensure that a problem we have identified is the root cause of a problem? The approach of asking five why questions to find the root cause is aimed at inferring the cause from the results (i.e., the problems). If we can follow the inferred cause step by step and obtain the same result (the problem), the cause in question can be identified as the root cause. Of course, the root cause must be consistent with the facts and the data collected from the workplace. The inferred root causes of our problem are in line with these principles (Table 3).

Any non-value-adding activity is classified as muda, a Japanese word meaning “waste” in English, which refers to anything that takes time but does not add value for our customers (patients). If we examine the root causes (Table 3) by reference to Toyota’s most famous seven types of common muda, it becomes clear that our process for transporting patients exhibits all 7 types of waste (6, 10). The waste of overproduction can be seen in the fact that the attendants always have a great deal of work to do at once, which could imply that the attendants do much more work than is necessary. The waste of waiting is evident in the fact that patients must wait 23 min for an attendant to pick up them and in the fact that an attendant must wait for a busy elevator to travel to the general ward. The waste of transportation can be seen in the fact that patients undergoing surgery and the public take the same elevator. The waste of processing results from the fact that the current process is not the best process, i.e., the current process has not been accurately rearranged and simplified. The waste of movement is apparent from the fact that the ward asks the attendant to deal with every difficulty immediately, whether the matter is large or small, and requires the attendant to continue walking among the various departments. The waste of stock on hand (inventory) is evident in the fact that patients who are supposed to have surgery on one day have their surgeries postponed until the following day due to delays in the delivery process. This situation prolongs the number of days that the patients spend in the hospital, creates medical disputes between doctors and patients and increases medical costs. If this situation also affects the patient’s safety and the quality of medical care the patient receives, prolongs the patient’s pain, causes the patient to distrust the doctor and the hospital, and causes disputes between the surgeon and the OR, these defects (flawed issues) tend to lead to medical controversies and disputes in the future (6, 8, 10, 12, 13).

Once we have identified the root causes of the problem, we can use this information to develop the countermeasures that are necessary to the task of eliminating these root causes. What can be done to eliminate the root cause of a problem and achieve a desired state? If many countermeasures could address the root cause, all of them should be listed. If the problem has more than one root cause, then for each root cause, we must formulate at least one countermeasure. The formulation of these countermeasures must be done in a process of brainstorming in accordance with the situation of the incident scene (6, 8, 13). There are several scientific ways of thinking that can help us formulate such countermeasures. Similar to an object, of which we can see the front, back, and sides, we can consider the root cause and the associated countermeasures from the front (i.e., the beneficial, effective, visible, and bright side of the issue), from the backside (i.e., the risks, costs, and all stakeholders, the invisible and dark side), and the sides (i.e., by adopting a perspective that ranges beyond the frontside and backside, including considerations such as the issue’s ecology, environment, and responsibility; we can observe the whole situation, including both its visible and invisible aspects). Analysis of a value stream map is one of the most important methods used to develop countermeasures. By analyzing the value stream map presented in Figure 3, which includes the time measured prior to improvement, we can see that the 23 min of attendant flow overlaps with the 19 min of the patient flow (Figure 5). Therefore, merely rearranging the process to change the position of the step of notifying the attendants can be predicted to decrease the amount of time a patient spends waiting for attendant movement in the existing process by 23 min, with the time now spent on this task being a mere 4 min. In further steps, we can use the ECRS (Eliminate, Combine, Rearrange, Simplify) technique (Figures 5, 6) to help formulate as many countermeasures as possible (14). We can also use the numerical data that we have measured to generalize our thinking and deduce actionable countermeasures (Figure 3) (6, 8, 10, 11). In addition, the most important point to bear in mind is that we are in the age of computer information technology, and so we must find ways to use information technology to help us develop countermeasures and solve problems (Figures 9, 11).

How can we decide which countermeasures to prioritize for implementation and which countermeasures should be implemented more slowly? From a practical perspective, we should first implement the countermeasures of which we and our department are capable. In general, countermeasures that can solve urgent problems, have a wide range of good effects, are low risk, be easily implemented, and are low-cost can be prioritized. In our case, if the process is changed only slightly, a strong time-saving effect can result (i.e., the change is ready to implement, effective, low cost, and low risk), so this change is listed as the highest priority. Problems related to the movement of attendants represent the bottlenecks (the most time-wasting step) for all delivery processes, so they are also listed as a top priority. Subsequently, the countermeasures with lower levels of priority should be implemented step by step because all the relevant countermeasures must be implemented to achieve the established goals (Table 3).

To determine whether the proposed countermeasures are effective, it is necessary to return to the workplace to observe and measure them. On this visit, we observed and measured 46 patients (Supplementary Figure 1). When the numerical data we obtained were included in the improved value stream map, we can see that it took approximately 20 min to send the patient to the OR (Figure 7). Figure 8 shows that all the indicators suggest that the targets have been achieved, and so all our countermeasures are effective. Based on the idea of continuous improvement and the upgrading of the hospital identification information system, the value stream map after improvement is more in line with future development and can evolve into a future process based on computer information-assisted patient and staff identification (Figure 9).

To determine whether this future process (Figure 9) is effective, it is necessary to return to the workplace once again for observation and measurement. On this occasion, we observed and measured 40 patients (Supplementary Figure 2). When the measured data were included in the value stream map after improvement, it becomes evident that it took approximately 19 min to send the patient to the OR (Figure 10). Although all these test results met our previously specified target value, we noticed that this time, the achievement rate (85.0%) of the total spent time was lower than the previous time (100%). This finding forces us to review the data shown in Supplementary Figure 2 once again, revealing that the reason for the lower achievement rate was mainly the amount of time that the patient spends preparing himself or herself for surgery in addition the time required for the patient to be transported to the nursing station. Based on patient-centered thinking, at this stage, we were not trying to shorten the amount of time the patient spends preparing himself or herself before surgery, and we will be careful to address this issue in the future. We conducted further analysis to determine that although the improved state indicated that the actual amount of time spent by the patient waiting for an attendant to bring her or him to the OR was 1 minute according to the value stream map after improvement (Figure 7) and 2 min with respect to the future process (Figure 10); these timeframes are close to meeting the requirements of the just-in-time principle but do not truly reach this benchmark (i.e., the just-in-time principle cannot be satisfied). According to the perspective of TPS, if we cannot satisfy the just-in-time principle, there continues to be waste in the workplace, and a new improvement project should be introduced (6, 12).

The ultimate focus of TPS is to insist on Kaizen, which is a Japanese word that, when translated into English, means continuous improvement, that is, improvement without an end point. As Taiichi Ohno noted, if we know that we should improve things for the better, we must insist on improving them until this task has been completed. This approach is the so-called soul of improvement (6). The word Kaizen has been included in many English dictionaries. Kaizen is described in the Cambridge Dictionary as “a Japanese way of running a company by always trying to improve the way people work and what they do.” The Oxford Learner’s Dictionary defines Kaizen as “the practice of continuously improving the way in which a company operates.” In summary, the Kaizen is a philosophy, a business management system, a mindset, and a methodology that focuses on continuous improvement. If the just-in-time principle cannot be satisfied, Kaizen must be implemented iteratively (6–8, 13).

Because the countermeasures that we provide can indeed solve our problems, the final step is to standardize our countermeasures so that all relevant colleagues have a common principle to follow. The countermeasures listed in Table 3 have been approved by the hospital president and must be followed and implemented by the whole hospital. Although acting in accordance with standard operating procedures is the best way for employees to do their work and although they are not blamed for doing things incorrectly, we believe that doing things in accordance with the appropriate standards is a minimum requirement for doing things well. Every time that we act, we should strive to do better than the standard, especially in the pursuit of patient safety and medical quality. When we act in accordance with the existing standards but cannot produce the results we expect, this situation indicates that another new problem has arisen. After we try to solve this new problem and after the problem is solved, a new standard is established, and this cycle is in line with the spirit of continuous improvement (6–8, 12, 13).

To ensure continuous improvement, we established four new targets without the aim of decreasing the time patients spent preparing themselves before surgery. Two targets regarding the total amount of time spent stipulate that the amount of time between when the ward notifies an attendant to send the patient to the OR to the time when the attendant receives the patient should be less than or equal to 30 min and that the achievement rate should be greater than or equal to 90%. The other two targets regarding attendant movement mandate that the total amount of time spent should be less than or equal to 14 min and that the achievement rate should be greater than or equal to 90%. Failure to meet these criteria should be managed as an abnormal event, which entails the automatic notification of hospital management by our computer information system. This automatic notification system is somewhat similar to Toyota’s autonomation, a term which refers to a machine that causes an automatic stop when defective products or abnormal events are detected. If more than half of the notifications of abnormal events result from the fact that the patient has taken too long to prepare himself or herself, then in the subsequent stage, we will address the events or procedures operative in the time between when the patient receives the notification from the ward to he or she is to be sent to the OR and the time when the patient travels to the nursing station.

Since the attendant has too many simultaneous responsibilities, which delays the delivery of patients to the operating room, can increasing the number of attendants solve this problem? This question is complicated by the fact that our hospital has an attendant quota; that is, each ward can only have one attendant. Therefore, increasing the number of attendants is not feasible (very difficult). Taiichi Ohno emphasized that the ultimate goal of TPS is to reduce costs, so in the operation of enterprises, the most streamlined distribution of manpower should be used to produce more products and complete work needs (6). Taiichi Ohno emphasized that the correct thinking is to ask what should be done before adding manpower; the first objective is to think about improving the workflow and the second is improving the equipment (6). Our way to improve the problems caused by the attendant quota is to organize and categorize the workflow for the attendants (5S), establishing the order in which attendants should prioritize their work and stipulating that the attendants have priority in the use of the elevators. We do not need to insist on the demand to increase the number of attendants to solve the problem. This approach is in line with the philosophy of the TPS, and we should continue to improve this approach in the future.

This article faces certain limitations. The first such limitation is that we could not satisfy the just-in-time principle, indicate the continuing presence of waste in our transporting process. The second limitation is that based on the root cause analysis, we are well aware that the provision of mutual support by attendants in their work is a very important form of interaction, but we still lack any way of developing norms to encourage the mutual support of attendants. The biggest reason proposed by the attendant head nurse for the failure of such support is the lack of a sufficient number of attendants to support each other. Thus far, we have no way of ensuring an appropriate distribution the workload of attendants. The third limitation is that the attendants joined the team after we had completed the Toyota management course and invited a teacher to guide us; accordingly, they did not participate in the formal course. Although we exerted our best efforts to teach them and communicate with them, they seemed to be able only to do what they were told; they were incapable of improving the content of their work and the environment in which they work.

In conclusion, the seemingly trivial process of patient transportation is associated with seven types of waste. The A3 8-step problem-solving process is useful for addressing such waste. Although our improved results have achieved the goals we established, they have not yet been perfected. We are deeply aware of the fact that process-related problems and elevator-related problems are relatively easy to solve, while attendant-related problems have always been the most difficult to address. In the process of improvement, we have learned and develop many concepts and forms of knowledge and have been nourished and cultivated by TPS. We are deeply aware of the fact that it is necessary to encourage the attendants to learn the concepts of TPS and lean thinking to ensure further improvement. As in the case of the philosophy of Toyota, quality people produce quality products. In the future, we will exert our best efforts to allow the TPS to nourish and cultivate attendants. We have the necessary confidence and enthusiasm to move forward toward continuous improvement and provide patients with added value.

## Data availability statement

The original contributions presented in this study are included in the article/Supplementary material, further inquiries can be directed to the corresponding author.

## Ethics statement

Ethical review and approval was not required for the study on human participants in accordance with the local legislation and institutional requirements. Written informed consent for participation was not required for this study in accordance with the national legislation and the institutional requirements.

## Author contributions

C-CL, J-WC, H-MC, Y-HC, and DC: protocol and project development, implementation of the protocol, manuscript revision, and accountable for all aspects of the work. C-CL, Y-HC, H-MC, and DC: data acquisition and interpretation. C-CL, J-WC, and DC: manuscript preparation. All authors read and approved the final manuscript.
