# Supplementary material for: Applying the Toyota production system to decrease the time required to transport patients undergoing surgery from the general ward to the operating room and reviewing the essence of lean thinking
Source: Front Med (Lausanne). 2022 Dec 7;9:1054583. doi: 10.3389/fmed.2022.1054583 (PMC9769121; doi:10.3389/fmed.2022.1054583)
Supplement: Supplementary file 1 [file Data_Sheet_1.PDF]

Supplementary Figure 1. The time points for measuring patient movement and the calculation of the time spent on each action.

Patient number	The time the patients and attendants are notified (A)	The time the patient arrives at nursing station (B)	The time the attendant arrives at nursing station (C)	The time the patient is given into the custody of the attendant (D)	The time the patient arrives at operating room (E)	Time spent transporting patient to the nursing station (B-A)	Time spent by the attendant reporting to the nursing station (C-A)	Time spent by patients waiting for the attendant to arrive (C-B)	Time spent on elevator transport (E-D)	Total amount of time spent (E-A)
1	10:23	10:31	10:34	10:34	10:40	8	11	3	6	17
2	14:00	14:08	14:08	14:08	14:15	8	8	0	7	15
3	14:12	14:15	14:16	14:17	14:27	3	4	1	10	15
4	7:30	7:40	7:45	7:45	7:55	0	15	5	10	25
5	8:56	9:02	9:00	9:03	9:05	6	4	0	2	9
6	7:40	7:40	7:50	7:50	7:51	0	10	10	1	11
7	7:50	7:55	7:55	8:00	8:05	5	5	0	5	15
8	9:45	9:54	9:56	9:56	10:15	9	11	2	19	30
9	10:18	10:22	10:25	10:25	10:27	4	7	3	2	9
10	10:50	10:55	11:05	11:05	11:10	5	15	10	5	20
11	12:30	12:40	12:45	12:45	12:48	10	15	5	3	18
12	13:18	13:30	13:34	13:35	13:38	12	16	4	3	20
13	13:15	13:21	13:22	13:22	13:30	6	7	1	8	15
14	14:20	14:30	14:35	14:40	14:42	10	15	5	2	22
15	16:45	16:50	16:52	16:52	16:55	5	7	2	3	10
16	18:00	18:12	18:16	18:20	18:21	12	16	4	1	21
17	8:50	9:00	9:03	9:05	9:10	10	13	3	5	20
18	9:40	9:45	9:52	9:55	9:57	5	12	7	2	17
19	10:35	10:45	10:45	10:49	10:50	10	10	0	1	15
20	11:20	11:29	11:31	11:34	11:40	9	11	2	6	20
21	14:00	14:06	14:05	14:07	14:15	6	5	0	8	15
22	14:40	14:53	14:50	14:55	14:56	13	10	0	1	16
23	7:45	7:46	7:48	7:50	8:05	1	3	2	15	20
24	8:10	8:15	8:17	8:20	8:25	5	7	2	5	15
25	11:30	11:40	11:46	11:46	11:50	10	16	6	4	20
26	8:44	8:51	8:52	8:55	9:00	7	8	1	5	16
27	8:35	8:43	8:45	8:47	8:50	8	10	2	3	15
28	8:40	8:50	8:50	8:50	8:55	10	10	0	5	15
29	9:22	9:25	9:24	9:25	9:30	3	2	0	5	8
30	11:00	11:17	11:15	11:20	11:25	17	15	0	5	25
31	13:50	13:57	13:58	13:59	14:05	7	8	1	6	15
32	14:05	14:10	14:10	14:15	14:22	5	5	0	7	17
33	15:45	15:50	15:52	15:53	15:55	5	7	2	2	10
34	7:40	7:45	7:47	7:50	7:55	5	7	2	5	15
35	8:50	9:08	9:01	9:09	9:10	18	11	0	1	20
36	12:50	12:53	12:52	12:58	12:59	3	2	0	1	9
37	8:00	8:23	8:23	8:25	8:30	23	23	0	5	30
38	12:11	12:16	12:20	12:20	12:35	5	9	4	15	24
39	13:58	14:00	14:10	14:10	14:15	2	12	10	5	17
40	8:00	8:15	8:15	8:15	8:16	15	15	0	1	16
41	7:30	7:45	7:45	7:50	8:00	15	15	0	10	30
42	7:30	7:40	7:45	7:48	7:50	10	15	5	2	20
43	7:30	7:45	7:45	7:50	7:55	15	15	0	5	25
44	10:49	10:53	10:52	10:55	10:56	4	3	0	1	7
45	14:23	14:25	14:24	14:30	14:31	2	1	0	1	8
46	10:12	10:14	10:15	10:20	10:21	2	3	6	1	9
Median (minutes)						7	10	2	5	16
