# Supplementary material for: Applying the Toyota production system to decrease the time required to transport patients undergoing surgery from the general ward to the operating room and reviewing the essence of lean thinking
Source: Front Med (Lausanne). 2022 Dec 7;9:1054583. doi: 10.3389/fmed.2022.1054583 (PMC9769121; doi:10.3389/fmed.2022.1054583)
Supplement: Supplementary file 2 [file Data_Sheet_2.PDF]

Supplementary Figure 2. The time points for measuring patient movement and the calculation of the time spent on each action based on our future process.

Patient number	Time the patients and attendants are notified (A)	Time the patient arrives at the nursing station (B)	Time the patient is picked up by an attendant (C)	Time the patient arrives at the operating room (D)		Time spent transporting patients to the nursing station (B-A)	Time spent by patients waiting for attendant to pick him or her up (C-B)	Time spent transporting patients from the ward to the operating room (D-C)	Total amount of time spent (D-A)
1	7:20	7:23	7:25	7:28		3	2	3	8
2	7:45	7:58	8:00	8:03		13	2	3	18
3	8:32	8:52	8:56	8:57		20	4	1	25
4	8:40	8:45	8:58	9:01		5	13	3	21
5	14:25	15:00	15:03	15:08		35	3	5	43
6	14:58	15:02	15:06	15:15		4	4	9	17
7	14:45	14:50	15:01	15:02		5	11	1	17
8	7:20	7:25	7:28	7:29		5	3	1	9
9	10:11	10:16	10:15	10:20		5	0	5	9
10	11:06	11:14	11:17	11:20		8	3	3	14
11	13:09	13:14	13:18	13:20		5	4	2	11
12	7:05	7:45	7:47	7:50		40	2	3	45
13	7:30	7:30	7:54	7:55		0	24	1	25
14	9:45	9:51	9:57	10:03		6	6	6	18
15	12:53	12:56	13:00	13:05		3	4	5	12
16	14:35	14:39	14:41	14:45		4	2	4	10
17	7:00	7:40	7:45	7:47		40	5	2	47
18	8:50	9:23	9:25	9:35		33	2	10	45
19	9:00	9:10	9:21	9:30		10	11	9	30
20	11:56	12:01	12:05	12:08		5	4	3	12
21	13:05	13:09	13:09	13:13		4	0	4	8
22	14:35	14:40	14:40	14:45		5	0	5	10
23	7:35	8:11	8:11	8:14		36	0	3	39
24	8:26	8:37	8:36	8:41		11	0	5	15
25	9:00	9:05	9:07	9:12		5	2	5	12
26	9:40	9:47	9:49	9:51		7	2	2	11
27	11:23	11:28	11:31	11:34		5	3	3	11
28	9:35	9:45	9:48	9:52		19	3	4	17
29	9:40	9:48	9:48	9:52		8	0	4	12
30	12:50	13:02	13:10	13:12		12	8	2	22
31	12:55	13:10	13:13	13:15		15	3	2	20
32	20:45	21:05	21:27	21:29		20	22	2	44
33	7:30	7:44	7:46	7:50		14	2	4	20
34	7:45	7:52	7:55	7:59		7	3	4	14
35	8:12	8:20	8:23	8:25		8	3	2	13
36	9:02	9:05	9:10	9:14		3	5	4	12
37	8:25	8:30	8:34	8:38		5	4	4	13
38	9:10	9:24	9:24	9:29		14	0	5	19
39	9:05	9:09	9:10	9:15		4	1	5	10
40	8:00	8:50	8:50	8:58		50	0	8	58
Median (minutes)	(* One elevator failure in the hospital during data collection)					7	3	4	15
